# Prognostic significance of peripheral immunity after thrombectomy in acute ischemic stroke: a retrospective study stratified by early post-EVT leukocyte count

**DOI:** 10.3389/fimmu.2026.1863605

**Published:** 2026-09-08

**Authors:** Tianfu Zhang, Chengzhou Huang, Peiran Zhang, Yapeng Guo, Junfeng Xu, Xiangjun Xu, Gaoshan Zhao, Shuting Yang, Zhiming Zhou, Xianjun Huang

**Affiliations:** Department of Neurology, Yijishan Hospital of Wannan Medical College, Wuhu, China

**Keywords:** acute ischemic stroke, endovascular thrombectomy, lymphocyte subsets, peripheral immune parameters, prognosis

## Abstract

**Background:**

The prognostic relevance of early peripheral immune profiles after successful endovascular thrombectomy (EVT) remains incompletely characterized. We evaluated whether associations between lymphocyte-subset percentages and 90-day functional outcome differed according to early post-EVT white blood cell (WBC) count.

**Materials and methods:**

This retrospective single-center study included acute anterior-circulation large-vessel occlusion who achieved successful reperfusion (mTICI 2b or 3) after EVT between January 2021 and December 2023. Blood samples obtained within 24 hours after EVT were classified as normal WBC (4.0–10.0 ×10^9/L) or elevated WBC (>10.0 ×10^9/L). The primary outcome was unfavorable 90-day functional status (mRS 3–6). Prespecified multivariable logistic models adjusted for age, sex, admission NIHSS, ASPECTS, ICA versus MCA occlusion, collateral status, and mTICI grade. Interaction, nonlinear, and sensitivity analyses were also performed.

**Results:**

A total of 334 patients met the eligibility criteria; after excluding one patient without a valid 90-day mRS assessment, 333 patients were included in the primary outcome analysis, including 205 (61.6%) in the normal-WBC group and 128 (38.4%) in the elevated-WBC group. In the normal-WBC group, lower CD19+ B-cell percentage (adjusted odds ratio [aOR] 0.918, 95% CI 0.864–0.976; P = 0.006) and higher NLR (aOR 1.170, 95% CI 1.043–1.311; P = 0.007) were associated with unfavorable outcome. In the elevated-WBC group, lower CD3+ T-cell percentage (aOR 0.960, 95% CI 0.926–0.995; P = 0.026) and lower CD8+ T-cell percentage (aOR 0.943, 95% CI 0.896–0.992; P = 0.024) were associated with unfavorable outcome. Biomarker-by-WBC interactions were significant for CD19, NLR, CD3, and CD8. Addition of these biomarkers numerically increased AUCs, but paired DeLong comparisons were not statistically significant.

**Conclusion:**

Early post-EVT WBC count modified the associations between selected peripheral immune-cell percentages and 90-day functional outcome. These exploratory findings require prospective validation.

**Trial registration:**

www.chictr.org.cn, identifier ChiCTR2300070650.

## Introduction

Endovascular thrombectomy (EVT) has transformed the management of acute ischemic stroke (AIS) caused by large-vessel occlusion (LVO), providing superior functional outcomes compared with medical therapy alone (1). Nevertheless, a substantial proportion of patients remain functionally dependent despite successful reperfusion, a phenomenon commonly termed futile recanalization (2, 3).

The mechanisms underlying futile recanalization are multifactorial and include clinical and imaging determinants as well as ischemia-reperfusion and thrombo-inflammatory injury (3–5). Accumulating evidence indicates that immune and inflammatory responses contribute to both secondary neuronal injury and subsequent tissue repair after ischemic stroke (6, 7). Disruption of the blood-brain barrier also facilitates interactions between resident glial cells and infiltrating peripheral leukocytes, with effects that vary according to cell type, timing, and tissue context (6–8). In successfully reperfused EVT cohorts, composite systemic inflammatory indices have also been associated with futile recanalization, supporting the relevance of systemic immune activation to post-EVT outcome (9).

Among readily available inflammatory biomarkers, postoperative NLR has been associated with functional outcome after mechanical thrombectomy (10, 11). Day-1 and dynamically measured NLR have also shown prognostic associations after reperfusion therapy, although optimal sampling times and thresholds vary across studies (12, 13). Fewer studies have concurrently evaluated multiple lymphocyte subsets (14). Peripheral lymphocyte-subset distributions may also change after AIS and have shown associations with clinical severity and prognosis (14). Therefore, we investigated the associations of early post-EVT lymphocyte-subset percentages (CD3+, CD4+, CD8+, CD19+, and CD56+CD3− NK cells) and NLR with 90-day functional outcome after successful EVT. We further evaluated whether these associations differed between patients with normal WBC counts (4.0–10.0 ×10^9/L) and those with elevated WBC counts (>10.0 ×10^9/L).

## Materials and methods

### Study design and patient population

This retrospective, single-center study analyzed consecutive patients who underwent EVT for anterior-circulation large-vessel occlusion at our comprehensive stroke center between January 2021 and December 2023. The study received approval from the Institutional Review Board (No. 20230031), which waived the requirement for written informed consent due to the retrospective design and complete data anonymization, minimizing participant risk.

Inclusion criteria encompassed: (1) age ≥18 years; (2) within 24 hours of symptom onset or last known well time; (3) admission National Institutes of Health Stroke Scale (NIHSS) score >6; (4) Alberta Stroke Program Early CT Score (ASPECTS) >5 on baseline non-contrast CT; (5) pre-stroke mRS score ≤2; (6) successful reperfusion defined as mTICI grade ≥2b.

**Figure 1:**
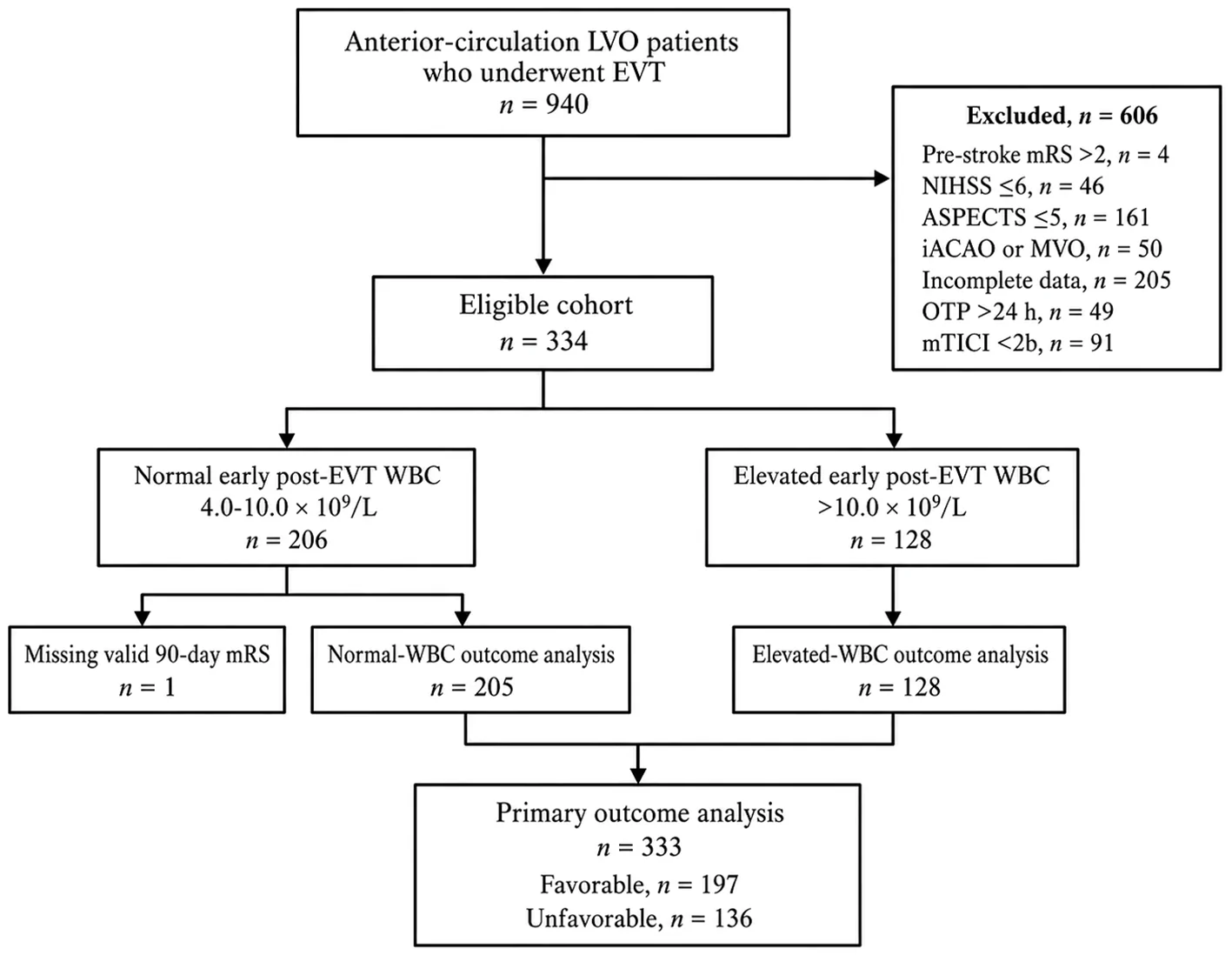
Flowchart. ASPECTS, alberta stroke program early CT score; LVO, large vessel occlusion; mRS, modified Rankin scale; EVT, endovascular treatment, NIHSS, national institutes of health stroke scale; mTICl, modified thrombolysis in cerebral infarction; MVO, multiple vessel occlusion; iACAO, isolated anterior cerebral artery occlusion.

Exclusion criteria were: (1) isolated anterior cerebral artery occlusion, or concurrent occlusions in multiple vascular territories; (2) absence of baseline complete blood count with differential and lymphocyte subset immunophenotyping within 24 hours after EVT. The flowchart of participants selection is **Figure 1**.

### Clinical and imaging data collection

Demographic and clinical characteristics were retrospectively extracted from medical records. Data included age, sex, vascular risk factors (hypertension, diabetes mellitus, atrial fibrillation, smoking, and alcohol use), admission NIHSS score, ASPECTS, occlusion site, collateral status, mTICI grade, and stroke etiology classified according to the Trial of Org 10172 in Acute Stroke Treatment (TOAST) criteria.

Collateral status was evaluated using the American Society of Interventional and Therapeutic Neuroradiology/Society of Interventional Radiology (ASITN/SIR) grading system (15), dichotomized into poor (grades 0-2) or good (grades 3-4) collateral circulation. DSA confirmed occlusion locations and assessed post-EVT reperfusion status using the mTICI scoring system. Images were independently analyzed by a board-certified neuroradiologist with over five years of experience. Both assessors were blinded to clinical outcomes and laboratory data.

### Laboratory parameters and immunophenotyping

Peripheral venous blood was obtained within 24 hours after EVT. WBC count was categorized as normal (4.0–10.0 ×10^9/L, inclusive) or elevated (>10.0 ×10^9/L). Because the elevated group represented leukocytosis rather than a combined leukocytosis/leukopenia category, the term “elevated WBC” is used throughout. Flow cytometry reported the relative percentages of CD3+ total T cells, CD3+CD4+ T cells, CD3+CD8+ T cells, CD19+ B cells, and CD56+CD3− NK cells; the CD4+/CD8+ ratio was calculated. Absolute lymphocyte-subset counts, counting-bead information, and sufficient single-platform data to reconstruct them were unavailable. Percentage-based findings were therefore interpreted as relative distributions and not as evidence of absolute cellular depletion or expansion.

### Outcome measures

The primary outcome was assessed using the mRS at 90 days. A favorable outcome was defined as mRS 0-2, and a poor outcome as mRS 3-6.

### Statistical analysis

Patients were categorized into favorable (mRS 0-2) and poor (mRS 3-6) outcome groups based on their 90-day mRS scores. Continuous variables were tested for normality using the Shapiro-Wilk test and, following confirmation of non-normal distribution, were expressed as median with interquartile range (IQR). Categorical variables were summarized as frequencies and percentages. Between-group comparisons utilized the Mann-Whitney U test or independent-samples T test for continuous variables and the chi-squared test or Fisher’s exact test for categorical variables, as appropriate.

The primary analyses used binary logistic regression with unfavorable 90-day outcome (mRS 3–6) as the dependent variable. To minimize data-driven variable selection, all primary models included a prespecified adjustment set comprising age, sex, admission NIHSS, ASPECTS, ICA versus MCA occlusion, collateral status, and mTICI grade (3 versus 2b). Separate models evaluated CD19 and NLR in the normal-WBC group and CD3, CD8, and NK-cell percentages in the elevated-WBC group. Results are reported as aORs with 95% CIs, together with complete-case sample sizes and event counts. Biomarker-by-WBC, biomarker-by-sex, and biomarker-by-mTICI interaction terms were tested in secondary analyses. A sensitivity analysis additionally adjusted the principal models for the absolute total lymphocyte count. ROC curves were generated for a common clinical base model and biomarker-augmented models within each WBC stratum; AUCs were compared using paired DeLong tests, and likelihood-ratio tests and Brier scores were also reported. No primary-outcome imputation was performed. Two-sided P<0.05 was considered statistically significant.

All statistical analyses were performed using SPSS version 27.0 (IBM Corporation, Armonk, NY, USA) and R software version 4.0.3 (R Foundation for Statistical Computing, Vienna, Austria). A two-tailed p-value < 0.05 was considered statistically significant.

## Results

### Patient baseline characteristics

A total of 334 patients met the eligibility criteria. One patient in the normal-WBC group lacked a valid 90-day mRS assessment and was excluded. The primary outcome cohort therefore comprised 333 patients, including 197 (59.2%) with favorable and 136 (40.8%) with unfavorable outcomes. The median age was 71 years (IQR 65–77), and 185 patients (55.6%) were male.

### Comparison of immune parameters between favorable and unfavorable outcome groups in the overall cohort

Patients with favorable outcomes (mRS 0–2) were significantly younger, more often male, had lower rates of atrial fibrillation, and were more frequently smokers compared to those with unfavorable outcomes (mRS 3–6). They also presented with lower baseline NIHSS scores, higher ASPECTS, a lower proportion of ICA occlusion, and better collateral status (**Supplementary Table 1**).

Regarding immune parameters, the favorable outcome group exhibited a significantly lower NLR (median 5.69 vs. 7.27, P < 0.001) and a higher CD19+ B‑cell percentage (median 11.90% vs. 10.50%, P = 0.035). No significant differences were observed for other lymphocyte subsets (CD3+, CD4+, CD8+, CD4+/CD8+ ratio, or NK cells) between the two outcome groups (all P > 0.05). Detailed comparisons are shown in **Supplementary Table 1**.

In overall-cohort models using the prespecified clinical adjustment set and additionally adjusting for WBC stratum, NLR remained associated with unfavorable outcome (aOR 1.067, 95% CI 1.005–1.134; P = 0.035), whereas CD19, CD3, CD8, and NK-cell percentages were not independently associated with outcome. Because the primary study question concerned effect heterogeneity by WBC status, the stratified and interaction analyses were considered the principal analyses. Detailed comparisons are shown in **Supplementary Table 2**.

### Stratified analysis by early post-EVT WBC count

Effect-modification analyses supported heterogeneity by early post-EVT WBC status. Biomarker-by-WBC interactions were significant for CD19 (P_interaction_=0.010), NLR (P_interaction_=0.034), CD3 (P_interaction_=0.028), and CD8 (P_interaction_=0.027), but not NK cells (P_interaction_=0.199).

Among 205 normal-WBC patients with valid outcome data, favorable outcome was associated with lower NLR (median 4.80 versus 6.71; P<0.001), higher CD19+ B-cell percentage (median 11.75% versus 9.95%; P = 0.008), younger age, lower admission NIHSS, higher ASPECTS, and less frequent ICA occlusion. The CD4+/CD8+ ratio did not differ significantly between outcome groups (**Table 1**). In prespecified multivariable models, lower CD19+ B-cell percentage (aOR 0.918 per percentage point, 95% CI 0.864–0.976; P = 0.006) and higher NLR (aOR 1.170 per unit, 95% CI 1.043–1.311; P = 0.007) were independently associated with unfavorable outcome.

**Table 1:** Baseline characteristics in the group with normal early post-EVT WBC counts.

Characteristics	Total	Favorable outcome	Unfavorable outcome	P value
	(n = 205)	(n = 125)	(n = 80)	
Demographics				
Age, median (IQR)	72 (66-77)	71(62.50-75)	74 (69.25-79)	0.001
Male, n (%)	101(49.3)	64(51.2)	37 (46.2)	0.489
DM, n (%)	37 (18.0)	21(16.8)	16 (20.0)	0.561
HTN, n (%)	129 (62.9)	78 (62.4)	51 (63.8)	0.845
AF, n (%)	119 (57.6)	67 (53.6)	51 (63.8)	0.151
Smoking, n (%)	41 (20.0)	26 (20.8)	15 (18.8)	0.720
Drinking, n (%)	43 (21.0)	27 (21.6)	16 (20.0)	0.784
Clinical characteristics				
NIHSS, median (IQR)	14 (12-17)	12 (10.5-16)	15.5 (12-18)	<0.001
ASPECT score, median (IQR)	9 (8-10)	9 (8-10)	8 (7-9)	<0.001
Occlusion site, n (%)				
ICA	64 (31.2)	32 (25.6)	32 (40.0)	0.030
MCA	141 (68.8)	93 (74.4)	48 (60.0)	
Good collaterals, n (%)	143 (69.8)	91 (72.8)	52 (65.0)	0.352
mTICI3, n (%)	147 (71.7)	94 (75.2)	53 (66.2)	0.165
TOAST, n (%)				
LAA	48 (23.4)	28 (22.4)	20 (25.0)	0.479
Cardioembolic	135 (65.9)	81 (64.8)	54 (67.5)	
Others	22 (10.7)	16 (12.8)	6 (7.5)	
Blood test				
NLR, median (IQR)	5.11 (3.79-7.09)	4.80 (3.46-5.96)	6.71 (4.31-8.63)	<0.001
TC, mmol/L, median± SD	4.15 ± 0.93	4.17 ± 0.94	4.14 ± 0.90	0.843
HDL, mmol/L, median (IQR)	1.37 (1.20-1.61)	1.35 (1.19-1.57)	1.38 (1.20-1.67)	0.327
LDL, mmol/L, median± SD	2.35 ± 0.78	2.39 ± 0.77	2.30 ± 0.79	0.455
CD3+ T-cell, %, mean ± SD	62.47 ± 12.50	63.44 ± 12.44	65.87 ± 12.28	0.174
CD4+ T-cell, %, mean ± SD	40.50 ± 9.54	40.50 ± 8.97	40.81 ± 10.53	0.815
CD8+ T-cell, %, median (IQR)	19.35 (14.43-24.40)	19.00 (14.35-23.35)	20.40 (15.03-26.85)	0.314
CD4+/CD8+, median (IQR)	2.07 (1.58-3.00)	2.12 (1.65-2.93)	1.97 (1.51-3.16)	0.449
CD19+ B-cell, %, median (IQR)	11.30 (7.65-15.25)	11.75 (8.28-15.93)	9.95 (6.10-13.68)	0.008
NK cell, %, median (IQR)	13.20 (8.90-20.03)	13.10 (9.55-19.75)	13.55 (7.63-21.24)	0.773

AF, atrial fibrillation; ASPECTS, Alberta Stroke Program Early CT Score; CAD, coronary artery disease;DM, diabetes mellitus; HTN, hypertension; HDL, high-density lipoprotein; IQR, interquartile range; ICA, internal carotid artery; LAA, large artery atherosclerosis; LDL, low-density lipoprotein; MCA, middle cerebral artery; NIHSS, National Institutes of Health Stroke Scale; NLR, neutrophil to lymphocyte ratio; SD, standard deviation; TC, total cholesterol; TOAST, Trial of Org 10172 in Acute Stroke Treatment.

Among 128 patients with elevated WBC counts, favorable outcome was associated with higher CD3+ T-cell percentage (median 65.75% versus 62.00%; P = 0.025), higher CD8+ T-cell percentage (median 21.90% versus 16.80%; P = 0.008), and lower NK-cell percentage (median 11.80% versus 13.65%; P = 0.046, **Table 2**). In the prespecified multivariable models, lower CD3+ T-cell percentage (aOR 0.960 per percentage point, 95% CI 0.926–0.995; P = 0.026) and lower CD8+ T-cell percentage (aOR 0.943, 95% CI 0.896–0.992; P = 0.024) were associated with unfavorable outcome. NK-cell percentage was not independently associated with outcome (aOR 1.034, 95% CI 0.991–1.079; P = 0.122; **Table 3**).

**Table 2:** Baseline characteristics in the group with elevated early post-EVT WBC counts.

Characteristics	Total	Favorable outcome	Unfavorable outcome	P value
	(n = 128)	(n = 72)	(n =56)	
Demographics				
Age, median (IQR)	70 (59.25-77)	68 (57.25-72.25)	74.50 (65-79.75)	<0.001
Male, n (%)	84(65.6)	58 (80.6)	26 (46.4)	<0.001
DM, n (%)	23 (18.0)	12 (16.7)	11 (19.6)	0.664
HTN, n (%)	85 (66.4)	48 (66.7)	37 (66.1)	0.944
AF, n (%)	63 (49.2)	27 (37.5)	36 (64.3)	0.003
Smoking, n (%)	57 (44.5)	42 (58.3)	15 (26.8)	<0.001
Drinking, n (%)	37 (28.9)	27 (37.5)	10 (17.9)	0.015
Clinical characteristics				
NIHSS, median (IQR)	14 (11-17)	13 (11-16)	15 (12-18)	0.006
ASPECT score, median (IQR)	9 (8-10)	9 (8-10)	9 (7-9)	0.016
Occlusion site, n (%)				
ICA	60 (46.9)	32 (44.4)	28 (50.0)	0.532
MCA	68 (53.1)	40 (55.6)	28 (50.0)	
Good collaterals, n (%)	82 (64.1)	57 (79.2)	25 (44.6)	<0.001
mTICI3, n (%)	82 (64.1)	50 (69.4)	32 (57.1)	0.150
TOAST, n (%)				
LAA	43 (33.6)	31 (43.1)	12 (21.4)	0.028
Cardioembolic	69 (53.9)	32 (44.4)	37 (66.1)	
Others	16 (12.5)	9 (12.5)	7 (12.5)	
Blood test				
NLR, median (IQR)	9.54 (6.60-14.39)	8.95 (6.35-13.41)	10.62 (6.98-15.57)	0.152
TC, mmol/L, median± SD	4.39 ± 1.16	4.33 ± 1.19	4.48 ± 1.12	0.479
HDL, mmol/L, median± SD	1.39 ± 0.33	1.37 ± 0.28	1.42 ± 0.38	0.444
LDL, mmol/L, median± SD	2.60 ± 0.97	2.60 ± 1.00	2.60 ± 0.92	0.985
CD3+ T-cell, %, median (IQR)	64.30(53.58-72.28)	65.75(56.95-72.95)	62.00(46.28-71.13)	0.025
CD4+ T-cell, %, mean ± SD	38.36 ± 9.98	39.64 ± 8.74	36.71 ± 11.25	0.099
CD8+ T-cell, %, mean ± SD	20.40 ± 8.69	21.86 ± 7.63	18.53 ± 9.64	0.031
CD4+/CD8+, median (IQR)	2.02(1.44-2.84)	1.97 (1.44-2.48)	2.30 (1.42-3.31)	0.142
CD19+ B-cell, %, median (IQR)	12.10 (7.83-17.68)	12.15 (7.38-17.78)	11.35(8.33-16.88)	0.766
NK cell, %, median (IQR)	12.85 (7.75-19.93)	11.80 (7.60-18.00)	13.65 (8.70-24.85)	0.046

AF, atrial fibrillation; ASPECTS, Alberta Stroke Program Early CT Score; CAD, coronary artery disease;DM, diabetes mellitus; HTN, hypertension; HDL, high-density lipoprotein; IQR, interquartile range; ICA, internal carotid artery; LAA, large artery atherosclerosis; LDL, low-density lipoprotein; MCA, middle cerebral artery; NIHSS, National Institutes of Health Stroke Scale; NLR, neutrophil to lymphocyte ratio; SD, standard deviation; TC, total cholesterol; TOAST, Trial of Org 10172 in Acute Stroke Treatment.

**Table 3:** Adjusted associations between peripheral immune parameters and unfavorable 90-day outcome according to early post-EVT WBC status.

Biomarker	Normal WBC N/events	Normal WBC OR (95% CI)	P value	Elevated WBC N/events	Elevated WBC aOR (95% CI)	P value	P for interaction
CD19+ B cells, %	205/80	0.918 (0.864-0.976)	0.006	128/56	1.024 (0.974-1.076)	0.361	0.010
NLR	205/80	1.170 (1.043-1.311)	0.007	128/56	1.013 (0.939-1.093)	0.745	0.034
CD3+ T cells, %	205/80	1.021 (0.993-1.050)	0.148	128/56	0.960 (0.926-0.995)	0.026	0.028
CD8+ T cells, %	205/80	1.012 (0.971-1.055)	0.566	128/56	0.943 (0.896-0.992)	0.024	0.027
NK cells, %	205/80	0.996 (0.960-1.034)	0.850	128/56	1.034 (0.991-1.079)	0.122	0.199

Adjusted odds ratios are reported per 1-percentage-point increase in lymphocyte-subset percentage and per 1-unit increase in NLR. All models adjusted for age, sex, admission NIHSS, ASPECTS, ICA versus MCA occlusion, collateral status, and mTICI grade (3 versus 2b). Complete-case analysis was used.

No significant sex interaction was observed for CD19, NLR, or CD3. A sex-by-CD8 interaction was observed in the elevated-WBC group (P_interaction_=0.023, **Supplementary Table 3**), with a stronger inverse association in women than in men; because the female subgroup contained 44 patients and CD8 showed evidence of nonlinearity, this finding may be considered exploratory.

No statistically significant biomarker-by-mTICI interaction was detected for CD19, NLR, CD3, or CD8 (**Supplementary Table 4**).

### Sensitivity analyses

After additional adjustment for the absolute total lymphocyte count, in the normal-WBC group, CD19 (aOR 0.921, 95% CI 0.866–0.979; P = 0.008) and NLR (aOR 1.297, 95% CI 1.084–1.551; P = 0.004) were independently associated with unfavorable outcome; in the elevated-WBC group, CD3 (aOR 0.956, 95% CI 0.922–0.992; P = 0.017) and CD8 (aOR 0.942, 95% CI 0.895–0.991; P = 0.022) were independently associated with favorable outcome (**Supplementary Table 5**).

### Predictive performance of immune parameters

**Figure 2:**
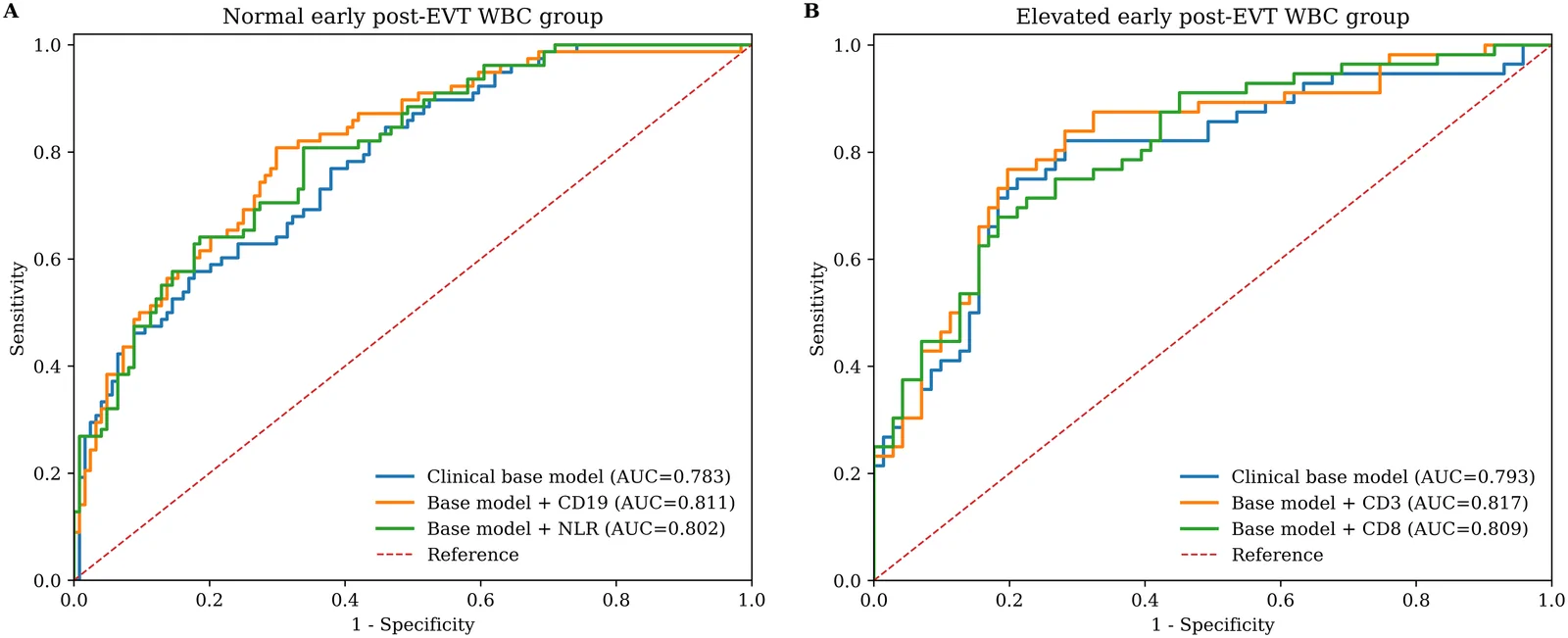
Receiver operating characteristic curves for clinical base models and biomarker-augmented models. **(A)** Normal-WBC group: clinical base model, base model plus CD19+ B-cell percentage, and base model plus NLR. **(B)** Elevated-WBC group: clinical base model, base model plus CD3+ T-cell percentage, and base model plus CD8+ T-cell percentage.

In the normal-WBC group, the common clinical base model had an AUC of 0.783 (95% CI 0.720–0.846). Addition of CD19 increased the AUC to 0.811 (95% CI 0.751–0.871; DeLong P = 0.060), whereas addition of NLR increased it to 0.802 (95% CI 0.742–0.862; DeLong P = 0.200). In the elevated-WBC group, the clinical base-model AUC was 0.793 (95% CI 0.711–0.874); addition of CD3 increased the AUC to 0.817 (95% CI 0.741–0.893; DeLong P = 0.128), and addition of CD8 increased it to 0.809 (95% CI 0.733–0.884; DeLong P = 0.414). Likelihood-ratio tests and Brier scores indicated improved overall model fit after biomarker addition, but none of the AUC differences was statistically significant by paired DeLong testing (**Figure 2**; **Supplementary Table 6**).

## Discussion

This study suggests that the prognostic significance of peripheral immune parameters following successful EVT was not uniform but differed according to early post-EVT WBC status. While previous studies have generally examined immune biomarkers across overall stroke populations (10–14), our analysis identified heterogeneity in biomarker–outcome associations in different WBC strata. In patients with normal early post-EVT WBC counts, CD19+ B-cell percentage and NLR were independently associated with functional outcome, whereas in those with elevated early post-EVT WBC counts, CD3+ and CD8+ T-cell percentages were independently associated with outcome. Formal interaction testing supported heterogeneity for these four biomarkers after adjustment for conventional clinical and imaging predictors. No significant biomarker-by-mTICI interaction was observed.

The most striking and counterintuitive finding of our study concerns CD8+ T cells in the elevated-WBC group. Experimental studies have implicated CD8+ T cells as potentially detrimental effectors in ischemic brain injury through cytotoxic mechanisms (16). However, in patients with elevated early post-EVT WBC counts, higher CD8+ T-cell percentages were independently associated with a lower probability of unfavorable outcome. This apparently paradoxical finding is compatible with emerging evidence of CD8+ T-cell functional heterogeneity after stroke. In a preclinical model, a distinct early-responder CD8+ regulatory-like subset adopted a neuroprotective effector state after entering the ischemic brain and limited inflammatory injury (17). Our findings therefore raise the possibility that the systemic inflammatory milieu may influence the functional state, trafficking, or peripheral distribution of CD8+ T cells. However, because functional phenotypes were not measured and only relative percentages were available, the present association cannot establish a direct protective effect or preservation of the absolute CD8+ T-cell number. Complementing this observation, NK-cell percentage was higher in patients with unfavorable outcomes in the unadjusted comparison but was not independently associated with outcome after multivariable adjustment. Experimental findings regarding NK cells are also context dependent: NK cells promoted acute tissue damage in one model (18), whereas CXCL12-recruited NK cells were protective in another (19). Thus, the present NK-cell result should not be interpreted as evidence of a causal detrimental effect. An exploratory sex-by-CD8 interaction suggested that the inverse association may have been stronger in women than in men, a finding that is biologically plausible in light of sex-related differences in post-stroke immune regulation (20) but requires validation because of the small female elevated-WBC subgroup. Together, these findings highlight the nuanced and context-dependent nature of post-stroke immunity.

The differential prognostic relevance of NLR across WBC strata extends previous literature on inflammatory biomarkers after stroke. Postoperative, day-1, and dynamically measured NLR have been associated with functional outcome after reperfusion therapy (10–13, 21). In our cohort, the association was present in patients with normal early post-EVT WBC counts but was not observed in those with elevated WBC counts, and the significant NLR-by-WBC interaction supported heterogeneity between the strata. These findings suggest that the prognostic relevance of NLR may depend on the broader leukocyte context rather than being uniform across all patients. When total WBC count remains within the normal range, NLR may more specifically reflect the relative balance between neutrophil mobilization and stroke-associated lymphocyte reduction (6, 7, 22). Conversely, when total WBC count is already elevated, NLR may reflect a more complex combination of stroke-related inflammation and other systemic inflammatory signals. NLR is also associated with post-stroke infection, which may reduce its specificity for cerebral ischemia-related immune responses (23). However, because the causes of leukocytosis were not systematically adjudicated in this retrospective dataset, these mechanistic explanations remain speculative.

The divergent prognostic significance of B cells across the two WBC strata further illustrates the complexity of post-stroke immune responses. In the normal-WBC group, a higher CD19+ B-cell percentage was independently associated with a lower probability of unfavorable outcome, consistent with the potential immunoregulatory relevance of B cells. Human studies have shown dynamic changes in circulating B-cell and regulatory lymphocyte distributions after AIS (24, 25), while experimental studies suggest that regulatory and IL-10-producing B cells can limit central and peripheral inflammation and improve neurological outcomes (26, 27). IL-10 may also improve stroke outcome by suppressing detrimental IL-17A responses (28). Nevertheless, the present findings do not establish a direct neuroprotective role of B cells. Because only relative lymphocyte-subset percentages were available, a higher CD19+ B-cell percentage cannot be assumed to represent preservation of the absolute B-cell count and may partly reflect reciprocal changes in other lymphocyte subsets. Additional adjustment for the absolute total lymphocyte count did not materially alter the association, but this sensitivity analysis cannot substitute for absolute B-cell counts or fully resolve the compositional limitations of percentage-based measurements. In the elevated-WBC group, CD19+ B-cell percentage was not independently associated with outcome, whereas CD3+ and CD8+ T-cell percentages were associated with outcome. The single early post-EVT measurement in our study cannot determine the temporal or functional mechanisms underlying this difference.

Our findings may have implications for immune-informed research in stroke, but they do not establish a basis for immunomodulatory treatment selection. Immune responses after stroke include potentially detrimental and reparative pathways, and broad immunomodulation may affect both (7). In the MARVEL randomized clinical trial, adjunctive methylprednisolone did not improve the primary 90-day disability outcome, although lower mortality and symptomatic intracranial hemorrhage rates were observed as secondary or safety outcomes (29). Our observational study did not assess biomarker-by-treatment interactions and therefore cannot determine whether peripheral immune heterogeneity contributed to those trial findings or identify a treatment-responsive immune subgroup. Moreover, the pleiotropic, cell- and tissue-specific actions of corticosteroids preclude attribution of their clinical effects to a single leukocyte population (30). Future prospective studies should incorporate pretreatment and serial post-treatment sampling, absolute lymphocyte-subset counts, functional immune phenotyping, systematic assessment of infection and other causes of leukocytosis, external validation, and prespecified biomarker-by-treatment interaction analyses. Because the immune measurements in the present study were obtained after EVT, our data cannot support pretreatment stratification. Whether immune-profile-guided therapy can improve outcomes requires evaluation in appropriately designed prospective interventional trials.

Several limitations warrant acknowledgment. First, the retrospective, single-center design may introduce selection bias and residual confounding despite consecutive patient enrollment. Second, lymphocyte subsets were available only as relative percentages, whereas absolute CD3+, CD8+, CD19+, and NK-cell counts were unavailable. Although the database contained the absolute total lymphocyte count and additional adjustment for this variable did not materially alter the principal associations, this analysis cannot substitute for absolute lymphocyte-subset counts. Third, immune measurements were obtained at a single early time point within 24 hours after EVT; pretreatment measurements and longitudinal immune profiling were unavailable, limiting our ability to characterize dynamic immune changes throughout recovery. Fourth, the causes of elevated WBC counts, including infection or other inflammatory conditions, were not systematically adjudicated. Fifth, we did not assess functional characteristics of immune cells, such as cytokine-producing capacity, activation markers, or regulatory and cytotoxic phenotypes, which limits mechanistic interpretation beyond relative cellular distributions. Sixth, the sex-stratified analyses were exploratory and may have been underpowered, particularly in women with elevated WBC counts. Finally, the cohort comprised anterior-circulation strokes, potentially limiting generalizability to posterior-circulation events and other clinical populations.

## Conclusions

Early post-EVT WBC count modified the associations between selected peripheral immune-cell percentages and 90-day functional outcome after successful thrombectomy. In patients with normal WBC counts, CD19+ B-cell percentage and NLR were associated with outcome, whereas CD3+ and CD8+ T-cell percentages were associated with outcome in patients with elevated WBC counts. The biomarkers improved overall model fit and numerically increased AUCs, although formal AUC comparisons were not statistically significant. These hypothesis-generating findings require prospective multicenter validation.

## Data Availability

The raw data supporting the conclusions of this article will be made available by the authors, without undue reservation.
