# Supplementary material for: Prognostic significance of peripheral immunity after thrombectomy in acute ischemic stroke: a retrospective study stratified by early post-EVT leukocyte count
Source: Front Immunol. 2026 Sep 8;17:1863605. doi: 10.3389/fimmu.2026.1863605 (PMC13593490; doi:10.3389/fimmu.2026.1863605)
Supplement: Supplementary file 1 [file DataSheet1.pdf]

frontiers

The International Society for the Advancement of Cytometry (ISAC) has highlighted the importance of including comprehensive methodological information to ensure data reproducibility and reliability. In line with this, Frontiers in Immunology now requires authors to submit a checklist for manuscripts that involve flow or mass cytometry. This checklist helps standardize the reporting process, improving the quality and transparency of published data. By doing so, we support scientific progress, making it easier for other researchers to replicate and validate experiments.

This form should be submitted with any manuscripts using flow or mass cytometry.

Sample/specimen/material description

- [ ] Total blood
- [x] PBMCs
- [ ] Organ digests

Other____

Did the samples suffer any treatment before or after incubation with the antibodies?

- [ ] Drug ____________________________________
- [ ] Cell permeabilization _______________________
- [ ] Dye _______________________________________
- [ ] Propidium iodine
- [x] Not applicable

Other ____

Instrument and antibodies

Name of the Cytometer Beckman Coulter Cytomics™ FC 500

**Antibodies and targets**	**Fluorochrome/ Metal**	**Catalog number/Company**
e.g. anti-CD4	FITC	Cat. XXX/ XXX ltd.
anti-CD3 (clone UCHT1)	PC5	Beckman Coulter / Immunotech; tetraCHROME CD45-FITC/CD4-PE/CD8-ECD/CD3-PC5, Cat. 6607013

anti-CD4 (clone SFCI12T4D11)	PE	Beckman Coulter / Immunotech;
anti-CD8 (clone SFCI21Thy2D3)	ECD	Beckman Coulter / Immunotech;
anti-CD19 (clone J3-119)	ECD	Beckman Coulter / Immunotech;
anti-CD56 (clone N901)	PE	Beckman Coulter / Immunotech;

Data analyses

1. Name of the software SPSS Statistics version 27.0; R version 4.0.3
2. Reference gating strategy in the manuscript or supplementary material

Gating strategy in (eg Figure X) Not available in the submitted manuscript or supplementary material
