# Supplementary material for: Prognostic significance of peripheral immunity after thrombectomy in acute ischemic stroke: a retrospective study stratified by early post-EVT leukocyte count
Source: Front Immunol. 2026 Sep 8;17:1863605. doi: 10.3389/fimmu.2026.1863605 (PMC13593490; doi:10.3389/fimmu.2026.1863605)
Supplement: Supplementary file 2 [file Table1.docx]

Table S1 Baseline characteristics in the overall-cohort

| Characteristics | Total | Favorable outcome | Unfavorable outcome | P value |
| --- | --- | --- | --- | --- |
|  | (n = 333) | (n = 197) | (n =136) |  |
| **Demographics**  Age, median (IQR) | 71 (65-77) | 69 (60-75) | 74 (69-79) | <0.01 |
| Male, n (%) | 185(55.6) | 122 (61.9) | 63 (46.3) | 0.003 |
| DM, n (%) | 60 (18.0) | 33 (16.8) | 27 (19.9) | 0.469 |
| HTN, n (%) | 214 (64.1) | 126 (64) | 88 (64.7) | 0.889 |
| AF, n (%) | 181 (54.4) | 94 (47.7) | 87 (64.0) | 0.003 |
| Smoking, n (%) | 98 (29.4) | 68 (34.5) | 30 (22.1) | 0.014 |
| Drinking, n (%) | 81 (24.0) | 54 (27.4) | 26 (19.1) | 0.082 |
| **Clinical characteristics** |  |  |  |  |
| NIHSS, median (IQR) | 14 (12-17) | 13 (11-16) | 15 (12-18) | <0.01 |
| ASPECT score, median (IQR) | 9 (8-10) | 9 (8-10) | 8 (7-9) | <0.01 |
| Occlusion site, n (%)  ICA | 124 (37.2) | 64 (32.5) | 60 (44.1) | 0.02 |
| MCA | 209 (62.8) | 133 (67.5) | 76 (55.9) |  |
| Good collaterals, n (%) | 225 (68.2) | 148 (75.5) | 77 (57.5) | <0.01 |
| mTICI3，n(%) | 229 (68.8) | 144 (73.1) | 85 (62.5) | 0.04 |
| TOAST, n (%)  LAA | 91 (27.3) | 59 (29.9) | 32 (23.5) | 0.212 |
| Cardioembolic | 204 (61.3) | 113 (57.4) | 91 (66.9) |  |
| Others | 38 (11.4) | 25 (12.7) | 13 (9.6) |  |
| **Blood test**  NLR, median (IQR) | 6.52 (4.38-9.75) | 5.69 (3.97-8.89) | 7.27 (5.25-11.31) | <0.01 |
| TC, mmol/L, median± SD | 4.39±1.16 | 4.23±1.04 | 4.28±1.00 | 0.479 |
| HDL, mmol/L, median (IQR) | 1.37 (1.17-1.61) | 1.36 (1.18-1.56) | 1.39 (1.15-1.67) | 0.280 |
| LDL, mmol/L, median (IQR) | 2.38 (1.85-2.92) | 2.35 (1.87-2.90) | 2.44 (1.85-2.99) | 0.986 |
| CD3+ T-cell，%, median (IQR) | 64.75（55.58-72.45） | 64.7（56.7-72.2） | 64.85（54.83-72.58） | 0.618 |
| CD4+ T-cell, %, mean ± SD | 38.36±9.98 | 40.18±8.88 | 39.12±10.76 | 0.294 |
| CD8+ T-cell, %, median (IQR) | 19.40 (14.48-24.63) | 19.80(14.90-24.45) | 18.35(13.75-25.30) | 0.324 |
| CD4+/CD8+, median (IQR) | 2.07(1.54-2.95) | 2.07 (1.56-2.80) | 2.04 (1.46-3.23) | 0.775 |
| CD19+ B-cell, %, median (IQR) | 11.37 (7.74-16.10) | 11.90 (7.98-17.40) | 10.50(6.80-14.58) | 0.029 |
| NK cell, %, median (IQR) | 13 (8.38-20.03) | 12.70 (8.30-19.10) | 13.55 (8.35-21.95) | 0.302 |

Abbreviations: AF, atrial fibrillation; ASPECTS, Alberta Stroke Program Early CT Score; CAD, coronary artery disease; DM, diabetes mellitus; HTN, hypertension; HDL, high-density lipoprotein; IQR, interquartile range; ICA, internal carotid artery; LAA, large artery atherosclerosis; LDL, low-density lipoprotein; MCA, middle cerebral artery; NIHSS, National Institutes of Health Stroke Scale; NLR, neutrophil to lymphocyte ratio; SD, standard deviation; TC, total cholesterol; TOAST, Trial of Org 10172 in Acute Stroke Treatment.

Table S2 The associations between peripheral immune parameters and outcome in the overall-cohort.

| Biomarker | N/events | aOR (95% CI) | P value |
| --- | --- | --- | --- |
| CD19+ B cells, % | 333/136 | 0.974 (0.940-1.011) | 0.166 |
| NLR | 333/136 | 1.067 (1.005-1.134) | 0.035 |
| CD3+ T cells, % | 333/136 | 1.003 (0.983-1.023) | 0.762 |
| CD8+ T cells, % | 333/136 | 0.992 (0.963-1.022) | 0.592 |
| NK cells, % | 333/136 | 1.013 (0.986-1.040) | 0.351 |

Abbreviations: ASPECTS, alberta stroke program early ct score; CI, confidence interval; NIHSS, National Institutes of Health Stroke Scale; NLR, neutrophil to lymphocyte ratio OR, odds ratio.

Each biomarker was entered in a separate model. Models adjusted for early post-EVT WBC group, age, sex, admission NIHSS, ASPECTS, ICA versus MCA occlusion, collateral status, and mTICI grade.

| WBC stratum | Biomarker | Male N/events | Male aOR (95% CI) | P value | Female N/events | Female aOR (95% CI) | P value | P for interaction |
| --- | --- | --- | --- | --- | --- | --- | --- | --- |
| Normal WBC | CD19+ B cells, % | 101/37 | 0.916 (0.833-1.006) | 0.066 | 104/43 | 0.920 (0.849-0.997) | 0.043 | 0.938 |
| Normal WBC | NLR | 101/37 | 1.150 (0.998-1.325) | 0.053 | 104/43 | 1.205 (0.995-1.458) | 0.056 | 0.703 |
| Elevated WBC | CD3+ T cells, % | 84/26 | 0.954 (0.913-0.997) | 0.038 | 44/30 | 0.971 (0.916-1.030) | 0.327 | 0.633 |
| Elevated WBC | CD8+ T cells, % | 84/26 | 0.973 (0.922-1.028) | 0.329 | 44/30 | 0.816 (0.707-0.941) | 0.005 | 0.023 |

Table S3. Sex-stratified analyses

Models included the biomarker-by-sex interaction term and adjusted for age, admission NIHSS, ASPECTS, ICA versus MCA occlusion, collateral status, and mTICI grade. The CD8 sex interaction is exploratory

Table S4. Reperfusion-grade-stratified analyses

| WBC stratum | Biomarker | mTICI 2b N/events | mTICI 2b aOR (95% CI) | P value | mTICI 3 N/events | mTICI 3 aOR (95% CI) | P value | P for interaction |
| --- | --- | --- | --- | --- | --- | --- | --- | --- |
| Normal WBC | CD19+ B cells, % | 58/27 | 0.936 (0.812-1.079) | 0.362 | 147/53 | 0.914 (0.855-0.978) | 0.009 | 0.768 |
| Normal WBC | NLR | 58/27 | 1.170 (0.940-1.455) | 0.159 | 147/53 | 1.170 (1.023-1.337) | 0.022 | 0.999 |
| Elevated WBC | CD3+ T cells, % | 46/24 | 0.963 (0.909-1.020) | 0.195 | 82/32 | 0.959 (0.918-1.001) | 0.057 | 0.904 |
| Elevated WBC | CD8+ T cells, % | 46/24 | 0.871 (0.785-0.965) | 0.009 | 82/32 | 0.970 (0.917-1.027) | 0.301 | 0.070 |

Models included the biomarker-by-mTICI interaction term and adjusted for age, sex, admission NIHSS, ASPECTS, ICA versus MCA occlusion, and collateral status.

Table S5. Sensitivity analyses additionally adjusted for absolute total lymphocyte count

| WBC stratum | Biomarker | N | Biomarker aOR (95% CI) | P value | Absolute lymphocyte count aOR (95% CI) | P value |
| --- | --- | --- | --- | --- | --- | --- |
| Normal WBC | CD19+ B cells, % | 205 | 0.921 (0.866-0.979) | 0.008 | 0.783 (0.410-1.494) | 0.458 |
| Normal WBC | NLR | 205 | 1.297 (1.084-1.551) | 0.004 | 2.066 (0.841-5.073) | 0.113 |
| Elevated WBC | CD3+ T cells, % | 128 | 0.956 (0.922-0.992) | 0.017 | 1.745 (0.695-4.380) | 0.236 |
| Elevated WBC | CD8+ T cells, % | 128 | 0.942 (0.895-0.991) | 0.022 | 1.493 (0.600-3.715) | 0.388 |

Models additionally adjusted for the absolute total lymphocyte count and the prespecified clinical covariates. This analysis does not substitute for absolute lymphocyte-subset counts.

Table S6. Discrimination, calibration, and model-comparison metrics

| WBC stratum | Model | N/events | AUC (95% CI) | AUC difference | DeLong P | Likelihood-ratio P | Brier score |
| --- | --- | --- | --- | --- | --- | --- | --- |
| Normal WBC | Clinical base model | 205/80 | 0.783 (0.720-0.846) | Reference |  |  | 0.181 |
| Normal WBC | Base model + CD19 | 205/80 | 0.811 (0.751-0.871) | 0.028 | 0.060 | 0.004 | 0.171 |
| Normal WBC | Base model + NLR | 205/80 | 0.802 (0.742-0.862) | 0.019 | 0.200 | 0.005 | 0.174 |
| Elevated WBC | Clinical base model | 128/56 | 0.793 (0.711-0.874) | Reference |  |  | 0.182 |
| Elevated WBC | Base model + CD3 | 128/56 | 0.817 (0.741-0.893) | 0.025 | 0.128 | 0.019 | 0.173 |
| Elevated WBC | Base model + CD8 | 128/56 | 0.809 (0.733-0.884) | 0.016 | 0.414 | 0.017 | 0.176 |

Within each WBC stratum, all models were fitted in the same complete-case sample. DeLong P values compare each augmented model with the clinical base model. Lower Brier scores indicate lower overall prediction error
